# Intramural Ectopic Pregnancy: Clinical Characteristics, Risk Factors for Uterine Rupture and Hysterectomy

**DOI:** 10.3389/fmed.2021.769627

**Published:** 2021-10-28

**Authors:** Xiaoran Chen, Lingyun Gao, Hongna Yu, Meijuan Liu, Shujun Kong, Sijian Li

**Affiliations:** ^1^Department of Ultrasound, The Affiliated Yantai Yuhuangding Hospital of Qingdao University, Yantai, China; ^2^Department of Obstetrics and Gynecology, The Affiliated Yantai Yuhuangding Hospital of Qingdao University, Yantai, China; ^3^Department of Obstetrics and Gynecology, Peking Union Medical College Hospital, Chinese Academy of Medical Sciences, Peking Union Medical College, Beijing, China

**Keywords:** intramural pregnancy, ectopic pregnancy, treatment, maternal outcomes, risk factors

## Abstract

**Background:** Intramural ectopic pregnancy is defined as the gestational sac (GS) is entirely within the myometrium, separate from the endometrial cavity and fallopian tubes, which is unsustainable and potentially life-threatening. The data investigating the clinical characteristics, management strategy, and fertility outcomes after treatment of intramural ectopic pregnancies are very limited due to its extreme rarity.

**Methods:** To investigate the clinical characteristics, treatment options, and fertility outcomes in patients with intramural ectopic pregnancy, a retrospective study included 56 patients was conducted. We also used logistic regression to identify potential risk factors for uterine rupture and hysterectomy in these patients.

**Results:** The mean age of patients was 31.1 years, with an average gestational age (GA) of 10.0 weeks, and the majority of the patient cohort (83.9%) had uterine or endometrial surgical history. 55.4% of the intramural pregnancy was diagnosed by preoperative imaging examination and 67.7% was detected by ultrasound. There was no dominant predisposed zone of the GS. Common treatment strategies included laparotomy surgery (41.1%) and laparoscopic surgery (35.7%), followed by methotrexate (7.1%) and expectant management (5.4%). Uterine rupture occurred in 9 patients and 8 patients underwent a hysterectomy, but no maternal demise was found. Logistic regression showed that a GA >10 weeks predicted a significantly higher risk of uterine rupture (Odds ratio [OR] 8.000, 95% confidence interval [CI] 1.456–43.966, *P* = 0.017) and hysterectomy (OR 12.333, 95% CI 2.125–71.565, *P* = 0.005), and GS located in the fundus also predicted higher probability of uterine rupture (OR 7.000,95% CI 1.271–38.543, *P* = 0.025). Among the ten patients who had a desire for fertility, 6 of them succeeded and 4 of them successfully delivered with a GA ≥ 34 weeks.

**Conclusion:** GA > 10 weeks was the risk factor for both uterine rupture and hysterectomy, while patients with GS located in the uterine fundus had a significantly higher risk of uterine rupture. The fertility outcomes were moderate after treatment. The management strategies should be individualized according to disease conditions and the desire for fertility, and early diagnosis is essential for optimizing clinical outcomes.

## Introduction

Intramural ectopic pregnancy is an extremely rare form of ectopic pregnancy that occupies <1% of ectopic pregnancies, and has only approximately 70 reported cases in the literature (1, 2). Intramural ectopic pregnancy occurs when the gestational sac is entirely within the myometrium, separate from the endometrial cavity and fallopian tubes; intramural pregnancy is unsustainable and potentially life-threatening (3–5). The manifestations of intramural ectopic pregnancy such as amenorrhea, lower abdominal pain, and vagal bleeding, are not specific and mimic the symptoms of ectopic pregnancy in other sites (2). These non-specific symptoms can sometimes lead to the misdiagnosis of intramural ectopic pregnancy as myoma, or choriocarcinoma (6). Besides, in previous studies, some patients experiencing intramural ectopic pregnancies reported no discomfort (7), and even the serum β-HCG was negative in one reported case (8). Due to the rarity of intramural ectopic pregnancy, data comprising mainly of case reports on the clinical characteristics, treatment options, and subsequent fertility outcomes in patients are limited.

Although the etiology of intramural pregnancy remains unclear, researchers have proposed potential mechanisms and several risk factors such as uterine surgery including dilation and curettage, cesarean section, or myomectomy (2, 9–11). Various diagnostic tools including ultrasound and MRI have been reported in previous studies (1, 12). Management strategies such as expectant treatment, methotrexate, laparoscopic surgery, and laparotomy surgery with or without fertility preservation have also been conducted (2). However, even with the rapid development of medicine, uterine rupture and hysterectomy still occurs (9, 11), which may lead to massive hemorrhage or permanent infertility. However, none of the current study evaluated the risk factors for uterine rupture or hysterectomy.

The objective of our study was to investigate the clinical characteristics, treatment options, and fertility outcomes in patients with intramural ectopic pregnancy, while also assess the potential risk factors for uterine rupture and hysterectomy.

## Materials and Methods

### Three Cases of Intramural Ectopic Pregnancy in Our Hospital

#### Demographic Data and Clinical Characteristics

This retrospective study was approved by the Ethics Committee of the Affiliated Yantai Yuhuangding Hospital of Qingdao University. We retrospective reviewed 3 cases of intramural ectopic pregnancy from 2017 to 2021. The mean age of these patients was 33.3 years (range 20–42), one of them was nulliparous. However, they all had uterine surgery such as cesarean section (case 3) or intrauterine operation (artificial abortion, cases 1 and 2). Two patients were within their first trimester and 1 patient was in her second trimester of 17 weeks.

All of them were preoperatively diagnosed with suspected intramural ectopic pregnancy through ultrasound; the GS was located at uterine fundus in one case and the GS was at the posterior wall of the uterus in the other two cases. Two patients presented vaginal bleeding at the time of admission, but there were no other symptoms. Before being admitted to our hospital, case 2 was diagnosed as ectopic pregnancy and received oral mifepristone and intramuscular methotrexate treatment. Case 3 received curettage and was given mifepristone since missed abortion was suspected. Case 1 underwent an artificial abortion 2 months before admission to our hospital (The clinical characteristics were listed in Table 1).

**Table 1:** Demographic data and clinical characteristics of patients with intramural ectopic pregnancy in our hospital.

**Patients**	**Age (y)**	**Obstertric history**	**Gestational age**	**Past surgical history**	**Symptoms; preoperative β-HCG**	**Features of ultrasonography related to intramural pregnancy**
1	20	G1P0	17 weeks + 2 days	Artificial abortion at 2 months ago	Vaginal bleeding; NA	The gestational sac was within the myometrium of the right posterior wall of the uterine, near the fundus. A live fetus was noted in gestational sac that had no communication with endometrial cavity, surrounded by myometrium
2	42	G4P1	7 weeks+	Artificial abortion 19 years ago; induced abortion at 2 years ago	None; 19,140 IU/L	The gestational sac was located in the left posterior wall of the uterine fundus, the muscular layer between the uterine cavity and gestational sac was about 5 mm in thickness
3	38	G2P1	10 weeks+	Cesarean section at 13 years ago	Vaginal bleeding; 521.9 IU/L	The gestural sac can be seen within the left posterior wall of the uterine, closed to the left corner of the uterus, without connected with the uterine cavity

#### Surgical Intervention and Related Treatment

**Figure 1:**
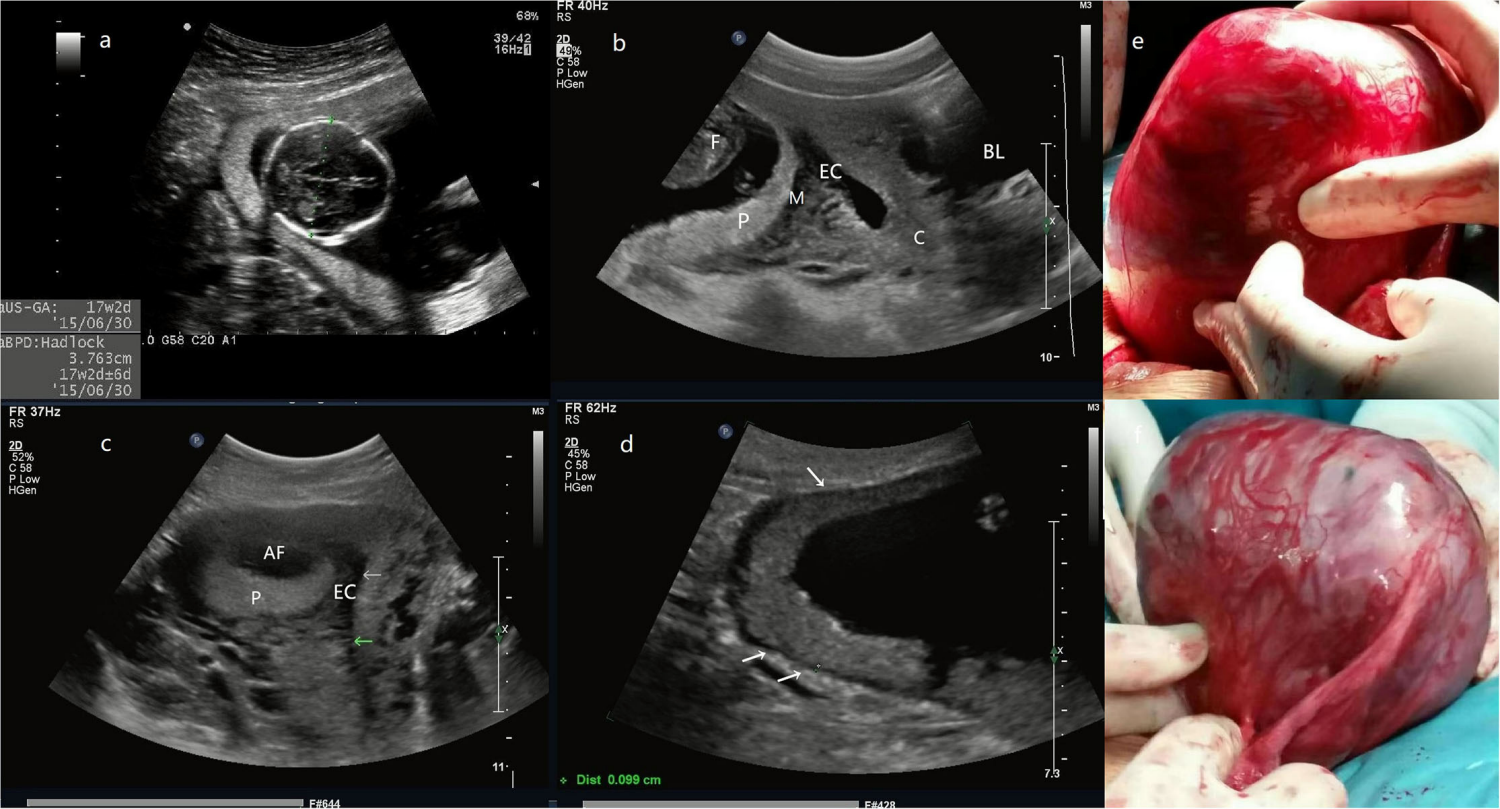
**(a)** the biparietal diameter of the fetus is 38 mm, the estimated gestational age is 17 weeks and 2 days. **(b)** The midsagittal section image reveals that the posterior myometrium is between GS and the EC. **(c)** The transversal section shows that the GS is located in the right posterior wall with a myometrial echo between GS and the EC (arrow). **(d)** The site of placental attachment covers the entire right posterior wall of the uterus, with a myometrial thickness of 0.9–1.2 mm (arrow). **(e)** The intraoperative image, an enlarged uterus with a thin wall mass (a size of 14 cm) protrudes at the right posterior wall of the fundus, the fetus, and fetal movement is also palpable. **(f)** Another photo during the surgery. The entire GS is within the myometrium and does not connect with the uterine cavity even after the removal of the fetus and the placenta (Patient 1, GS, gestational sac; F, fetus; P, placenta; M, myometrium; EC, endometrial cavity; AF, amniotic fluid; C, cervix; BL, bladder).

**Figure 2:**
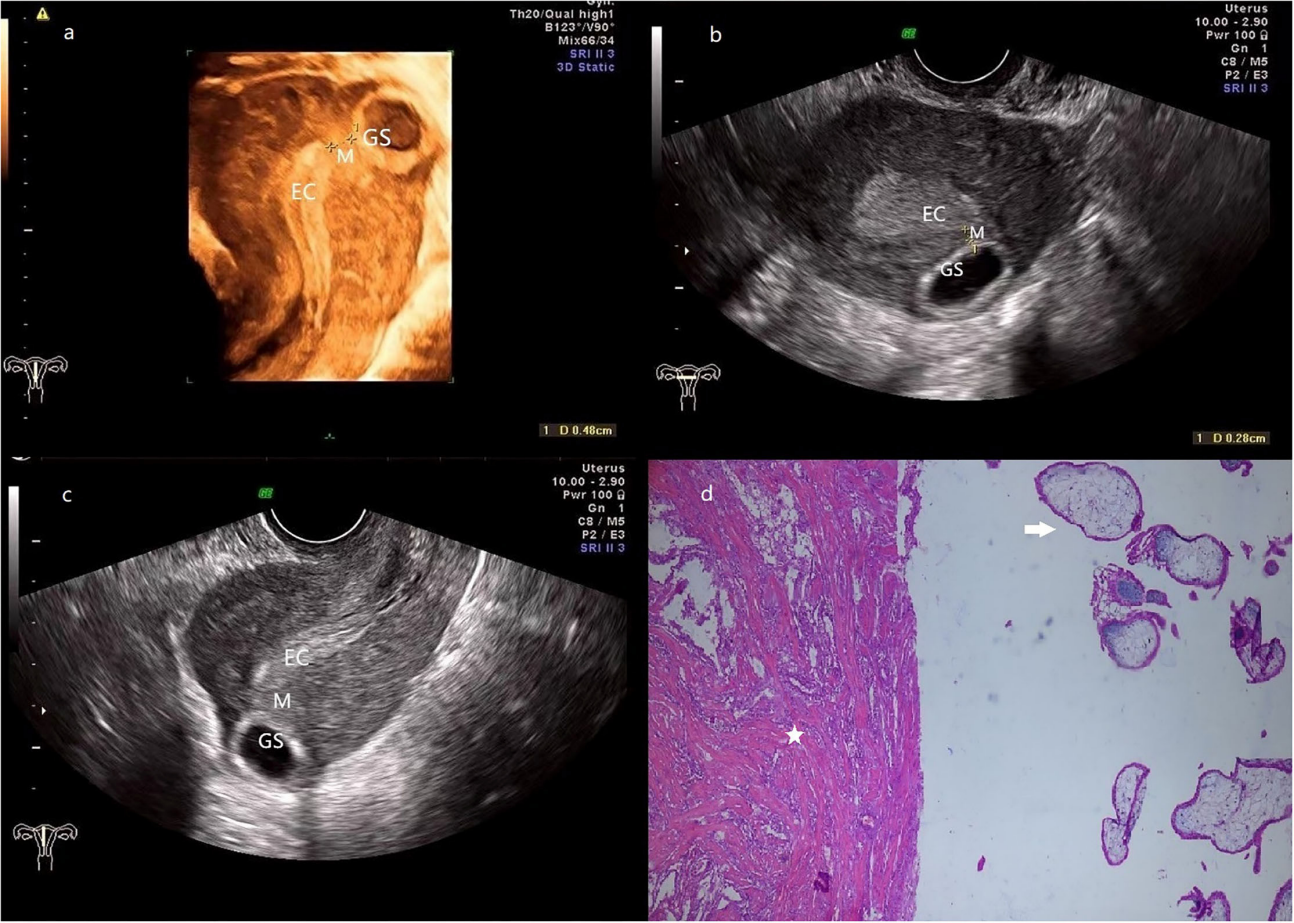
**(a)** 3-D ultrasonography reveals the relative location of EC, GS (a size of 2.7*1.0 cm), and the myometrium (0.5 cm in thickness) is located between them. **(b,c)** transversal **(b)** and sagittal **(c)** section image showed that the GS is located at the left posterior wall of the fundus, and the connection with EC is disrupted by the myometrium. **(d)** The pathologic examination demonstrates that muscular tissue (star) coexists with the chorionic villus (arrow). (Patient 2, GS, gestational sac; M, myometrium; EC, endometrial cavity).

**Figure 3:**
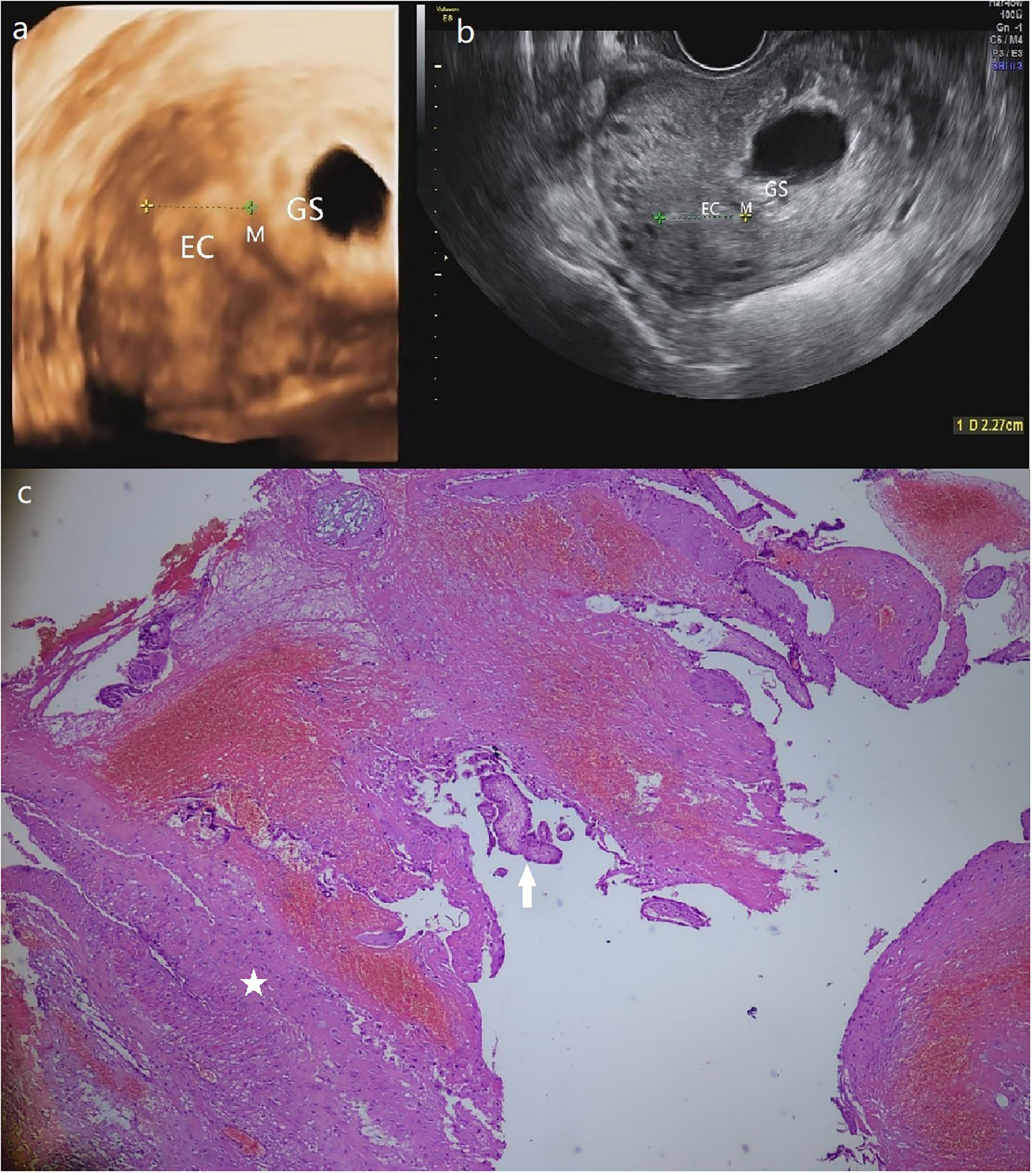
**(a)** The GS in a size of 4.9*3.4 cm is detected in a heterogeneous echo mass of 6.1*4.5 cm at the left posterior uterine wall near the left corner of the 3-D ultrasound. **(b)** Transversal section image in 2-D ultrasound demonstrates the intramural pregnancy and shows that the GS has no communication with EC. **(c)** The pathological result shows the myometrium (star) and the chorionic villus (arrow). (Patient 3, GS, gestational sac; M, myometrium; EC, endometrial cavity).

After suspected diagnosis of intramural ectopic pregnancy, surgical management was planned. Two patients in the first trimester received minimally invasive surgery and the third one underwent laparotomy. Moreover, fertility-sparing surgery with complete conceptus removal was conducted in all three patients, regardless of whether they had the desire to preserve fertility. The intraoperative diagnosis confirmed the intramural ectopic pregnancy in all 3 patients but no uterine rupture was detected. One patient (case 1) was given abdominal aorta balloon occlusion due to the potentially life-threatening hemorrhage indicated by the presence of abundant blood vessels on ultrasonography and the large size of the gestational sac (Figure 1). The surgery in all three patients was successful with a mean estimated intraoperative blood loss of 216.7 ml, no blood transfusion or hysterectomy occurred. The postoperative pathological results again confirmed the diagnosis of intramural pregnancy; chorionic villus was within the myometrium (case 2 and 3, Figures 2, 3), and there was no communication between the gestational sac and the endometrial cavity.

#### Results of Follow-Up

The postoperative recovery was uneventful in all 3 patients and they were discharged with a mean time of 5 days post-surgery. The subsequent postoperatively pelvic ultrasonography revealed no abnormality at the 6-month follow-up. Although their fertility was well-preserved during the surgery, the patients did not have a desire to conceive till the follow-up (Table 2).

**Table 2:** Surgical treatment, postoperative recovery, and fertility outcomes of patients with intramural ectopic pregnancy in our hospital.

**Patients**	**Surgical options; intraoperative blood loss**	**Other treatment**	**Hospitalization time after surgery**	**Outcomes and follow-up**
1	Laparotomy, hysterotomy, enucleation of conceptus, and hysteroplasty; diagnostic curettage/300 ml	Abdominal aorta ballon oclusion	5 days	Discharged uneventfully; not desire to fertility till follow-up
2	Laparoscopic exsicion of conceptus/150 ml	Mifepristone 25 mg bid for 3 days and intramural methotrexate 50 mg before admission	4 days	Discharged uneventfully; not desire to fertility till follow-up
3	Laparoscopic exsicion of conceptus; hysteroscopy/200 ml	Curettage 17 days before admission, combined with intermittent mifepristone 50 mg bid for 5 days	4 days	Discharged uneventfully; not desire to fertility till follow-up

### Literature Review

To collect all available English language data, we performed a systematic literature review of studies on intramural ectopic pregnancy published between 1960 and 2021. The keywords used for searching in PubMed, Embase, and Scopus were as follows: “intramural pregnancy”; “intramural ectopic pregnancy”; “intramyometrial pregnancy”; “uncommon ectopic pregnancy.” Relevant references cited within these articles were also reviewed. The exclusion criteria included patients with ectopic pregnancy in other sites, cases reported by letters or personal opinions, non-English literature, reports with no demographic and treatment information. Otherwise they were enrolled in analysis. After the screening process We selected 53 cases of intramural ectopic pregnancy reported in 38 studies (The detailed inclusion process can be found in Supplementary Figure 1). Finally, we generated a database that included demographic features, clinical and treatment characteristics of these 56 cases.

Clinical characteristics were analyzed to identify independent variables that might predicted spontaneous uterine rupture, including maternal age, prior uterine surgical history (<2 times, ≥2 times), methods of conception (natural conception or assisted reproductive technology), gestational age at presentation (≤ 10, >10 weeks), preoperative serum β-HCG level (IU/L), diagnosis (preoperative, intra, or postoperative) and location of the gestational sac (fundus or other location of the uterus). Prior uterine surgical history referred to surgeries that may damage the structure of myometrium or endometrium, such as curettage, artificial or induced abortion, cesarean section, myomectomy, etc. Previously mentioned variables and uterine rupture (yes or no) were assigned to evaluate the impact on hysterectomy.

### Statistical Analysis

Normally distributed continuous variables are described by means ± standard deviation (range), otherwise, they are presented as medians and interquartile ranges (IQRs). Counts (percentages) are used to express discrete variables. Categorical variables were compared by the chi-squared test. Univariate analysis was performed to assess risk factors for uterine rupture and hysterectomy. Factors with *p* < 0.2 were subjected to multivariate analysis using the logistic regression model to identify independent risk factors. The area under the receiver operating characteristic (ROC) curve (AUC) was used to evaluate the predictive performance of the identified risk factors. A two-tailed *p* < 0.05 was considered significant. Statistical analysis was conducted using SPSS (Version 21.0; SPSS Inc., Chicago, IL, USA).

## Results

The mean age of patients was 31.1 years (range: 19–42), with an average gestational age of 10.0 weeks (range: 5–30), and most of them (83.9%) had uterine or endometrial surgical history. The most common uterine surgery was curettage, with 35 patients (62.5%) experiencing curettage at least one time; the second most common surgery was cesarean section (12 patients, 21.4%). The preoperative serum β-HCG level was available in 38 patients but the value was variable, ranging from negative to 87474.0 IU/L, with a mean level of 18240.4 IU/L.

More than half (55.4%) of the cases had been preoperatively diagnosed with intramural ectopic pregnancy, mainly by ultrasound (67.7%), and 10 cases were confirmed by MRI. Twenty five patients were diagnosed intraoperatively or confirmed by pathology. The location of GS varied with no dominant predisposed zone; gestational sacs were located within the posterior wall (35.7%), the uterine fundus (30.4%), or within the lateral and anterior wall of the uterus. Treatment options often administrated laparotomy surgery (41.1%) and laparoscopic surgery (35.7%), followed by methotrexate (7.1%) and expectant management (5.4%). Of the 20 patients who received laparoscopic surgery, 6 were treated with combination of hysteroscopy. Similarly, in those treated with laparotomy surgery, two women were implemented aorta balloon occlusion, and another 2 patients experienced uterine artery embolization. Other options were less common with 3 patients receiving surgical enucleation and 1 patient receiving methotrexate plus potassium intracapsular injection. Moreover, simple bilateral uterine arteries embolization or hysteroscopy surgery was conducted in two patients. Most of them underwent conceptus removal to preserve fertility, and no maternal demise was reported. All of them recovered uneventfully after treatment (Table 3).

**Table 3:** Clinical characteristics, treatment, and maternal outcomes of the database.

**Variables**	**Percentile**	**Variables**	**Percentile**
Age (y)	31.1 ± 6.6	Location of gestational sac	*N* = 56
Previous uterine surgery	*N* = 51	Fundus	17 (30.4%)
No or <2 times	28 (50.0%)	Anterior wall	7 (12.5%)
≥2 times	23 (41.1%)	Posterior wall	20 (35.7%)
Gestational age (weeks)	10.0 ± 5.4 (54)	Left side of the uterus	6 (10.7%)
≤ 10	39	Right side of the uterus	6 (10.7%)
>10	15	Methods of treatment	*N* = 56
Methods of Conception	*N* = 56	Expectant treatment	3 (5.4%)
Nature	7 (12.5%)	Methotrexate	4 (7.1%)
ART	49 (87.5%)	Laparoscopic surgery	20 (35.7%)
Serum β-HCG level (IU/L)	18249.4 (38)	with hysteroscopy	6
Diagnosis time	*N* = 56	Laparotomy surgery	23 (41.1%)
Preoperative	31 (55.4%)	with UAE/UA ligation	1/1
Intra or postoperative	25 (44.6%)	with ABO	2
Diagnosis methods	*N* = 56	Surgical enucleation	3 (5.4%)
US	21	Methotrexate + potassium	1
MRI	10	UAE	1
Operation or pathology	25	Hysteroscopy	1
Intraoperative blood loss (ml)	483.0 (27)	Hysterectomy	8 (14.3%)
Blood transfusion	3 (50)	Uterine rupture	9 (16.1%)
Fertility outcomes	*N* = 17		
Not desire to fertility	7	Pregnancy (before delivery)	2
Desire to fertility	10	Successfully delivery	4
Failure	3	Term birth	3
Success	6	Preterm birth	1
Preparing	1	Recurrence of IEP	1

*NA, not applicable; HCG, human chorionic gonadotrophin; US, ultrasound; MRI, magnetic resonance imaging; UAE, uterine artery embolization; ABO, aorta balloon occlusion; IEP, intramural ectopic pregnancy*.

Uterine rupture was the most threatening emergency in the reported cases and was documented in 9 patients (16.1%). Potential risk factors that may predict uterine rupture are listed in Supplementary Table 2a. Gestational age (*P* = 0.010) and location of the gestational sac (*P* = 0.017) were identified by univariate analysis and further evaluated by multivariate logistic regression. Gestational age >10 weeks (Odds ratio [OR] 8.000, 95% confidence interval [CI] 1.456–43.966, *P* = 0.017) and location of the gestational sac in the fundus (OR 7.000, 95% CI 1.271–38.543, *P* = 0.025) remained statistically significant. The ROC curve demonstrated that AUC for gestational age >10 weeks and location of gestation sac in fundus was 0.733 (95% CI 0.540–0.927, *P* = 0.028), 0.716 (95% CI 0.523–0.910, *P* = 0.041), respectively. Moreover, using the cut point of gestational age as 10 weeks had a sensitivity of 66.7% and specificity of 82.2% in predicting uterine rupture. A combination of these two factors yielded a better predictive performance with an AUC of 0.793 (95% CI 0.596–0.989, *P* = 0.006) (Supplementary Figures 2a–c).

Although fertility preservation was successful in most cases, 8 patients (14.3%) underwent hysterectomy. The factors that may impact the probability of hysterectomy are listed in Supplementary Table 3. In univariate analysis, gestational age over 10 weeks and uterine rupture significantly increased the chance of hysterectomy. However, in subsequent multivariate regression, only gestational age over 10 weeks predicted a significantly higher risk of hysterectomy (OR 12.333, 95% CI 2.125–71.565, *P* = 0.005), with an AUC of 0.777 (95% CI 0.590–0.965, *P* = 0.013) (Supplementary Figure 2d). Similarly, we set the cut-off value at 10 weeks of gestational age for predicting hysterectomy, showing that the sensitivity and specificity were 75.0, 80.4%, respectively.

Subsequent pregnancy outcomes were available in 17 patients whose fertility was preserved. Seven of them did not have a desire for fertility and ten prepared for conception. One patient was preparing for IVF-ET and three patients failed to be pregnant. Among them, one experienced spontaneous abortion and another failed to conceive after IVF-ET. Moreover, one patient received IVF-ET, 5 months later, but that attempt resulted in a chemical pregnancy. Seven months later, she got pregnant by IVF-ET but intramural pregnancy was again detected and was terminated by laparoscopic resection of conceptus and hysteroplasty. The remaining six patients succeeded to conceive and 4 of them successfully delivered with GA ≥ 34 weeks, 2 of them are currently pregnant.

## Discussion

This current study presented the largest cohort emphasizing on the clinical characteristics, treatment options, and fertility outcomes in patients with intramural ectopic pregnancy. Moreover, we firstly evaluated the risk factors for uterine rupture and hysterectomy in this rare type of ectopic pregnancy. This may add new insight and help to improve the management of this disease.

Uterine rupture is a severe gynecologic emergency in intramural ectopic pregnancy which usually leads to massive obstetric hemorrhage (4, 5, 11, 13, 14). The average blood loss in patients who experienced uterine rupture was 1428.6 ml, which was significantly higher than those who had not experienced this complication (152.0, *P* < 0.001). Our study revealed a relatively high incidence of uterine rupture of 16.1%; early identification of risk factors for uterine rupture could definitely optimize the risk stratification and subsequent management in this population. Our research demonstrated that gestational age > 10 weeks and gestational sac located in the uterine fundus indicated a 7 to 8 times higher risk of uterine rupture, compared with those who had a lower gestational age or a different location of the gestational sac. A more advanced gestational age commonly represents a larger gestational sac, given that the gestational sac is completely surrounded by myometrium, rather than the endometrial cavity. The ever-growing conceptus confined to the myometrium may cause a pressing rupture, which may ascribe to the pulling force of the unusual myometrial vasculature or the thinner covering of the myometrium (15). We also noted that the mean gestational age in patients who embedded in the fundus was significantly greater compared with the patients whose conceptus was implanted in other site (12.3 vs. 9.1 weeks, *P* = 0.044), which partially interpreted that the location of the gestational sac significantly impacts the risk of rupture. Meanwhile, the myometrial structural strength, elasticity, and malleability of the fundus may be inferior compared with the other sites of the uterus becasue of the distribution of the uterine muscular layer (16). This result emphasizes the importance of an early diagnosis of intramural ectopic pregnancy and an accurate determination of the gestational sac's location. Patients with advanced gestational age or fundal location of gestational sac should receive timely interventions.

Hysterectomy eliminates the possibility of further fertility after treatment, which also greatly impacts the psychophysiological conditions of patients, especially those in the childbearing period. We found that a gestational age > 10 weeks was an independent risk factor for hysterectomy, which was consistent with the pregnancy physiology that uterine blood flow increases as the gestational age increases (17). In addition, hysterectomy is the ultimate method to treat uncontrolled maternal hemorrhage (18). The probability of hysterectomy has decreased in recent years (75% of hysterectomy reported before the 2010s), which may be attributed to early diagnosis and progress in management. Some patients in their second trimester were treated with a combination of aorta balloon occlusion or uterine artery embolization (7, 12). Hence, it may be advisable that patients with a high risk of hysterectomy develop a pre-arranged plan and undergo a more comprehensive preoperative evaluation and procedures such as uterine artery embolization, uterine artery ligation, and aorta balloon occlusion.

The diagnosis and treatment of intramural ectopic pregnancy have been gradually established and improved. Transvaginal ultrasonography, especially 3D ultrasound enables accurate localization of the gestational sac and plays an important role in the diagnosis of intramural ectopic pregnancy (1). Our study supported this method of diagnosis since all the three cases of intramural pregnancy in our hospital were correctly diagnosed by ultrasonography preoperatively, and nearly half of the published cases were also diagnosed through ultrasound. In patients with complicated or atypical echoic features, MRI may be a favorable supplement to clearly demonstrate the relationship between gestational sac and endometrial cavity (12, 19). We propose the use of transvaginal ultrasonography with 3D imaging for screening and MRI as a reserve technique in suspected cases of intramural ectopic pregnancy, in consideration of the cost and efficiency.

Expectant treatment, methotrexate administration, surgical enucleation, and minimally invasive or laparotomy surgery have been used to treat patients with intramural pregnancy in previous studies but there is no adequate comparison between these different methods due to the rarity of intramural pregnancy. Therefore, individualized management strategies have been suggested, mainly based on the disease conditions and patients' desire to preserve fertility (20). Expectant treatment should be only preserved in those with very low HCG level or who show signs of spontaneous abortion and presents no discomfort or mild clinical manifestation (21). Methotrexate can be used based on the guideline of ectopic pregnancy, and surgery is required if treatment fails (4, 19). Shen et al. suggested that laparoscopic surgery could be performed alone without hysteroscopy in most cases of early intramural ectopic pregnancy, whose safety and efficiency had been verified (10). However, we emphasize that in patients with advanced gestational age, laparotomy should be preferred, especially for patients in their second trimester, because they may experience massive hemorrhage and the size of the uterus may not be suitable to perform laparoscopic surgery. In some patients with earlier gestational age and gestational sac protruded to the uterine cavity, hysteroscopic treatment may be more suitable and without abdominal incision. Moreover, uterine artery embolization and aorta balloon occlusion may be effective in reducing the hemorrhage and increase the chance of fertility preservation (7, 22, 23), and can be combined with other treatment strategies. A comprehensive and objective explanation about the advantages and disadvantages of a variety of treatment options should be carried out when managing patients with intramural pregnancies.

There were only two cases of intramural ectopic pregnancy with fetal survival had been reported and the gestational ages were 30, 37 weeks, with the latter case reported by letter (5, 24). However, they survived till the late trimester because both cases were misdiagnosed as normal intrauterine pregnancies. Nonetheless, these two cases experienced life-threatening maternal hemorrhage and were treated with hysterectomy. The rest of the patients experienced inevitable fetal wastage during the first or second trimester. Since most of the patients who suffered intramural pregnancy were of childbearing age, concerns about the impact of this disease and its treatment on subsequent pregnancy outcomes should be highlighted. In our research, successful delivery with gestational age ≥ 34 weeks has been reported in 4 patients, another 2 patients are currently pregnant, with an overall pregnancy rate of 60% in those who have a desire for fertility. Besides, two patients conceived but one of them experienced spontaneous abortion and the other one experienced an ectopic pregnancy. This demonstrated a relatively satisfactory pregnancy outcome after fertility-sparing treatment. Surgical removal of conceptus with preservation of uterus should be recommended in those who have a desire for fertility. But uterine surgery itself predisposes patients to intramural pregnancy, so accurate screening needs to be performed to exclude intramural ectopic pregnancy during future conceptions since recurrence of intramural pregnancy has been observed (23).

Several limitations of our study must be stated. Since most cases are reviewed from the literature, the heterogeneity of this study cannot be ignored. Secondly, the rarity of this disease makes it difficult to conduct a more effective analysis. Thirdly, there was no pregnancy outcome data in most of these patients, so the impact of treatment options on fertility outcomes could not be accurate evaluated. Finally, we excluded cases from letters and non-English literature, and we did not have institutional assess to some articles, which may bias the results. Due to its rarity, more research on intramural pregnancy is warranted.

## Conclusion

Gestational age > 10 weeks was the risk factor for both uterine rupture and hysterectomy, while patients with gestational sac located in the uterine fundus had a significantly increased risk of uterine rupture. The fertility outcomes were moderate after fertility-sparing treatment. The management strategies should be individualized based on the disease conditions and the desire for fertility, and early diagnosis is essential to optimize the clinical outcomes.

## Data Availability Statement

The original contributions presented in the study are included in the article/Supplementary Material, further inquiries can be directed to the corresponding author/s.

## Ethics Statement

This retrospective study was approved by the Ethics Committee of the Affiliated Yantai Yuhuangding Hospital of Qingdao University (reference number: YTYH2019-399). Written informed consent for publication of their clinical details and/or clinical images was obtained from the patients. A copy of the consent form is available for review by the Editor of this journal.

## Author Contributions

XC and LG wrote the manuscript and completed the work of follow-up. HY and ML participated in the literature review and statistical analysis. SK completed the surgeries and treatment strategies, and conceived and designed the study. SL participated in conception and design of the study, modifying the manuscript. All authors read and approved the manuscript.

## Conflict of Interest

The authors declare that the research was conducted in the absence of any commercial or financial relationships that could be construed as a potential conflict of interest.

## Publisher's Note

All claims expressed in this article are solely those of the authors and do not necessarily represent those of their affiliated organizations, or those of the publisher, the editors and the reviewers. Any product that may be evaluated in this article, or claim that may be made by its manufacturer, is not guaranteed or endorsed by the publisher.

## Supplementary Material

Supplementary Figure 1 The detailed inclusion process is presented in the PRISMA flow diagram.

Supplementary Figure 2 The ROC curve revealed the AUC for identifying risk factors for uterine rupture and hysterectomy. **(a)** The performance of gestational age (GA) >10 weeks in predicting uterine rupture; **(b)** The impact of the gestational sac (GS) located in the uterine fundus on uterine rupture; **(c)** the AUC of the combination of GA > 10 weeks and GS located in the fundus; **(d)** The predictive performance of GA > 10 weeks in hysterectomy.

Supplementary Table 1 Database of our study.

Supplementary Table 2 **(a)** Univariate and multivariate analysis of clinical outcomes (uterine rupture vs. not rupture); **(b)** The predictive performance of the identified risk factors for uterine rupture.

Supplementary Table 3 Univariate and multivariate analysis to identify the potential risk factors for hysterectomy.
