# Supplementary figures and images for: Intramural Ectopic Pregnancy: Clinical Characteristics, Risk Factors for Uterine Rupture and Hysterectomy
Source: Front Med (Lausanne). 2021 Oct 28;8:769627. doi: 10.3389/fmed.2021.769627 (PMC8583088; doi:10.3389/fmed.2021.769627)

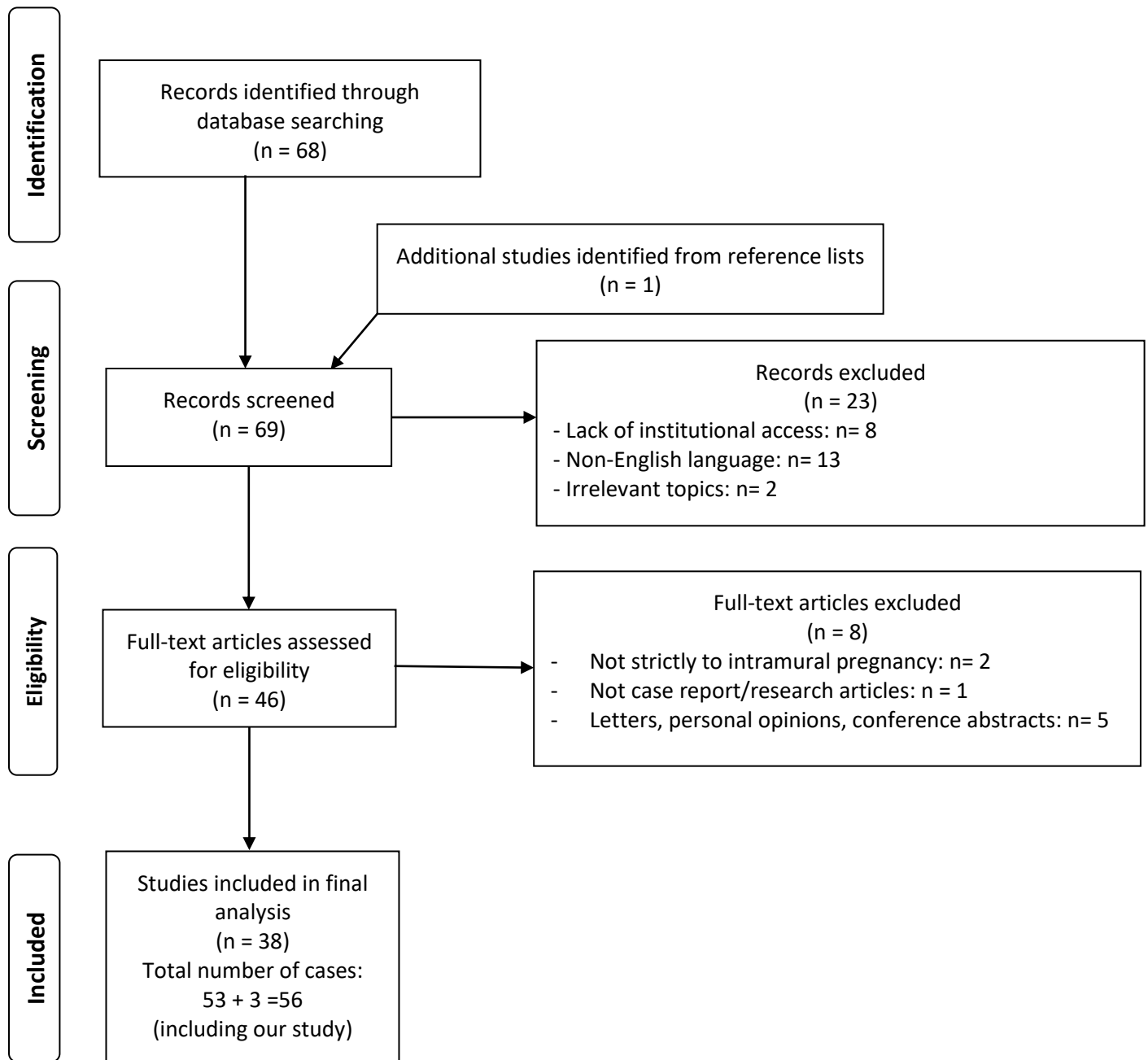

Supplement: Supplementary Figure 1 — The detailed inclusion process is presented in the PRISMA flow diagram. [file Data_Sheet_1.PDF]

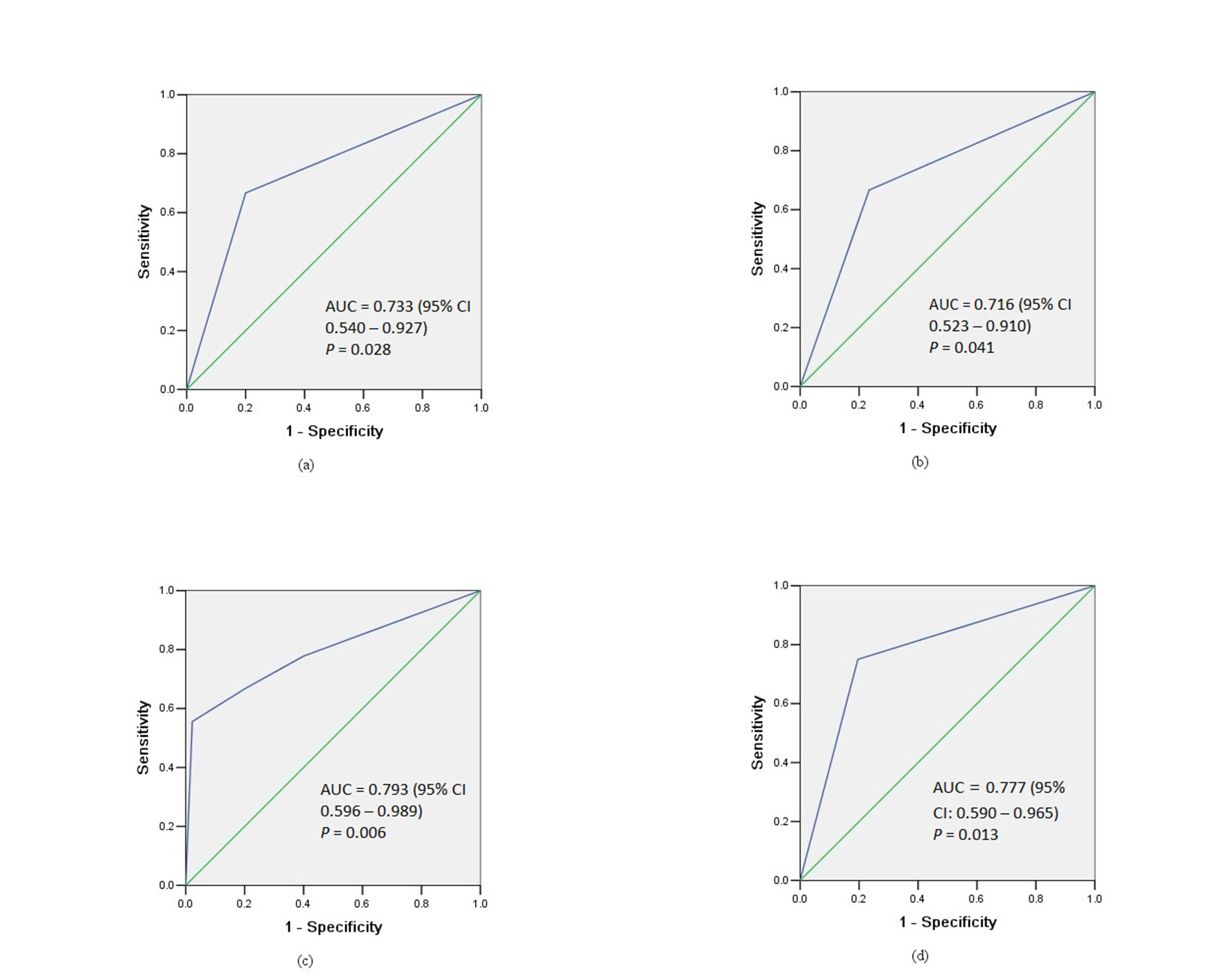

Supplement: Supplementary Figure 2 — The ROC curve revealed the AUC for identifying risk factors for uterine rupture and hysterectomy. (a) The performance of gestational age (GA) >10 weeks in predicting uterine rupture; (b) The impact of the gestational sac (GS) located in the uterine fundus on uterine rupture; (c) the AUC of the combination of GA > 10 weeks and GS located in the fundus; (d) The predictive performance of GA > 10 weeks in hysterectomy. [file Image_1.JPEG]
