# Supplementary material for: Intramural Ectopic Pregnancy: Clinical Characteristics, Risk Factors for Uterine Rupture and Hysterectomy
Source: Front Med (Lausanne). 2021 Oct 28;8:769627. doi: 10.3389/fmed.2021.769627 (PMC8583088; doi:10.3389/fmed.2021.769627)
Supplement: Supplementary Table 1 — Database of our study. [file Table_1.DOCX]

Supplementary table S1. Database of our study.

| Reference | No. | Age (y) | Obstetric/ Surgical history | Gestational age at diagnosis | Diagnosis method | Location of gestation sac / lesion size (cm) | treatment | Arterial embolism | Uterine rupture | Subsequent pregnancy |
| --- | --- | --- | --- | --- | --- | --- | --- | --- | --- | --- |
| McGowan Laryy (1965) | 1 | 28 | G1P0; curettage 2 times (with fundal perforation at 1st curettage) | 14weeks+ | postoperative pathology | fundus of the uterus; 10cm fetus | total abdominal hysterectomy | N | Y | NA |
|  | 2 | 24 | G1P0; curettage (with fundal perforation during surgery) | 10weeks+ | postoperative pathology | fundus of the uterus; 6(5cm fetus) | laparotomy excision of the conceptus | N | N | NA |
| Emil F. Cava et al. (1978) | 3 | 29 | G3P2; NA | 20weeks+ | postoperative pathology | within the right fundal wall of the uterus; 16cm fetus | total abdominal hysterectomy | N | Y | NA |
| Fait Gary et al. (1987) | 4 | 20 | G2P0; curettage at 12 weeks | 30weeks+ | postoperative pathology | fundus of the uterus; 1270g fetus, survival | exploratory laparotomy, total abdominal hysterectomy | N | Y | NA |
| Kenneth A. Ginsburg et al. (1989) | 5 | 33 | G9P2; curettage 5 times | 16weeks | US | anterior and medial to the right cornual region; NA | total abdominal hysterectomy | N | Y | NA |
| Steven W. Tucker (1995) | 6 | 36 | G5P2; NA | 8weeks | postoperative pathology | posterior wall; 6 | laparoscopic excision of conceptus | N | N | NA (artificial abortion; induced abortion) |
| Hsin Fen Lu et al. (1997) | 7 | 37 | G4P1A2; curettage 2 times, cesarean section | 5weeks | US | the right posterior uterine wall; 3.8*1.4*2.0 | total abdominal hysterectomy | N | N | NA |
| Farah W Lone et al. (2001) | 8 | 40 | P7; curettage; endometrial biopsy | 8+weeks | postoperative pathology | left uterine wall; 3*2.5 | total abdominal hysterectomy | N | N | NA |
| Helene B Bernstein et al. (2001) | 9 | 35 | G3A2; curettage | 6weeks | US; confirmed by laparoscopy | left uterine wall; 2 | expectant management | N | N | spontaneous abortion |
| Mohammad Ashraf et al. (2003) | 10 | 36 | NA; IVF | NA | laparoscopy | posterior wall of the uterine | None | N | N | continue to pregnancy to second trimester |
| Vasilos Dousias et al. (2003) | 11 | 31 | G3P1A2; curettage 2 times | NA | postoperative pathology | uterine fundus; 4.3*4*2.5 | laparotomy lesion excision | N | N | NA |
| Gui Se Ra Lee et al. (2003) | 12 | 25 | G1P0; RSO for right teratoma torsion | 6w+5d | 3D TVU | right lateral side of the myometrium, below the right cornu and above the uterosacral ligament; 1.5 | Laparotomy and lesion resection | N | N | NA |
| Hyun Sun Ko et al. (2006) | 13 | 30 | G4P1A3; curettage 3 times and 1 cesarean section | 13weeks | MRI | lower anterior uterine wall; 4.5*3.6*3.1 | intramural methotrexate (50mg/m2) | N | N | NA |
|  | 14 | 28 | G6P0; curettage 6 times | 9weeks | MRI | posterior body of the uterus; 2.8*1.8*1.8 | intramural methotrexate (50mg/m2) | N | N | NA |
| Won I. Park et al. (2006) | 15 | 35 | G1; laparotomy myomectomy; IVF | 37days after ET | US | the lfet posterior uterine wall; NA | laparoscopic excision of conceptus | N | N | IVF-ET 92days after surgery and uneventfully delivered by cesarean section at 38 weeks |
| Adel Al-Nazer et al. (2009) | 16 | 37 | G4P3; three Cesarean sections | 10 weeks +3d | TVU | intramural pregnancy near the Cesarean scar, anterior wall; 4.57*4.36 | Laparotomy and lesion resection | N | N | NA |
| Salih Taskın et al. (2009) | 17 | 28 | G4P3; N | 7 weeks | TVU | anterior cervical lip; NA | curettage; enucleation | N | Y | NA |
| Tracy Glass et al. (2010) | 18 | 29 | G1P0; N | 7weeks | TVU+MRI | The posterior-fundal portion of the uterus; 7.1*6.1 | curettage; laparoscopic diagnostic; laparotomy enucleation | N | Y | NA |
| Clara Ong et al. (2010) | 19 | 36 | G1P0; N | 6 weeks | TVU (2D +3D) | the posterior myometrium of the uterus equidistant from the two uterine cornua; 1.4 | injection of potassium and methotrexate | N | N | NA |
| Hiroshi Nabeshima et al. (2010) | 20 | 38 | G3P1; left salpingectomy for EP and a cesarean section | 8 weeks | TVU | Fundal; 3 | laparoscopic remove of conceptus | N | N | NA |
| Teruo Ohtsuka et al. (2010) | 21 | 22 | G1P0; curettage | 6 weeks 5 days | TVU +MRI | the anterior muscular layer of the uterine cervix; 2.5 | methotrexate + abdominal surgical removal 7 days later; bilateral uterine arteries ligation | N | N | NA |
| Z. Bouzari et al. (2010) | 22 | 28 | G3A2; curettage 2 times | 26weeks | intraoperative | the fundus and posterior aspect of uterus; NA | laparotomy surgical excision of conceptus | N | Y | NA |
|  | 23 | 32 | G2A1; curettage | 6 weeks | US; confirmed by MRI | the posterior myometrium; NA | intramural methotrexate (50mg/m2) | N | N | NA |
| Anis Fadhlaoui et al. (2011) | 24 | 38 | G5P1A2; salpingectomy | 13weeks | MRI | the fundal uterine wall; NA | laparotomy and enucleation of conceptus | N | Y | NA |
| Shaoguang Wang et al. (2013) | 25 | 28 | G2P0; abortion | 6 weeks | MRI | approximately the midline of the fundus uterus; NA | bilateral uterine arteries embolization | Y | N | NA |
| Emma Kirk et al. (2013) | 26 | 25 | G4P1; cesarean section; curettage | 5 weeks 6 days | intraoperative; TVU suspected to tubal EP | Posterior uterine wall; 2 | laparoscopic examination; laparotomy resection and repair | N | Y | NA |
| Kimberly Bannon et al. (2013) | 27 | 27 | G1P0; myomectomy; curettage | 10 weeks | 3D TVU and CT | posterior left lateral wall of the uterus; 6 | methotrexate; laparoscopic enucleation | N | N | NA |
| Pei-Ju Wu et al. (2013) | 28 | 20 | G3P0; 2 curettage | 8 weeks | intraoperative | the right fundal myometrium; 3 | hysteroscopy; laparoscopic enucleation; methotrexate | N | N | NA |
| M. MEMTSA et al. (2013) | 29 | 38 | G5P3; curettage | 8 weeks | pathology; postoperative US | myometrium of the left posterior uterine wall; NA | conservative management | N | N | Normal pregnancy 2y later; preterm birth at 34weeks |
| M. MEMTSA | 30 | 33 | G1P1; cesarean section; curettage | retained product of pregnancy (5 weeks after CS) | US | posterior uterine wall; NA | evacuation of retained product | N | N | Normal pregnancy 1y later; Spontaneous delivery at term |
| M. MEMTSA | 31 | 38 | G1P0; N | 9weeks | biopsy; US; laparoscopy | posterior uterine wall; NA | methotrexate 25mg injection | N | N | NA |
| M. MEMTSA | 32 | 37 | G1P0; N | 11weeks | US | deep in the myometrium of the left uterine wall; NA | continue to pregnancy and suspected rupture at 20weeks, emergency hysterectomy | N | N | NA |
| Yi Wang et al. (2015) | 33 | 21 | G3P1; two curettage | 8 weeks | intraoperative laparoscopy | the left fundal myometrium; 2 | laparoscopic resection | N | N | NA |
| Shuhong Li et al. (2016) | 34 | 20 | G1P0; curettage | 18.5weeks | TVU; MRI | the right posterior lateral side of the uterus fundus; 14*14 | transfemoral aorta balloon occlusion; laparotomy excision gestational sac | N | N | NA |
| Na-na Liu et al. (2017) | 35 | 34 | G2P1; laparoscopic surgery for endometriosis; curettage | 8 weeks | TVU; hysteroscopy | the posterior wall; 3.7 | laparoscopic resection | N | N | NA |
| Na-na Liu et al. (2017) | 36 | 19 | G1P0; curettage | 13weeks | intraoperative laparoscopy | the left posterior wall; 4.2 | laparoscopic resection | N | N | NA |
| Na-na Liu et al. (2017) | 37 | 40 | G4P0; curettage | 8 weeks+ | TVU; hysteroscopy | the right side of the uterine wall; 5 | laparoscopic resection | N | N | NA |
| Long Kong et al. (2017) | 38 | 20 | G1P0; curettage | 17weeks+2d | TVU; MRI | the myometrium in the right posterior wall of the uterine; NA | UAE and laparotomy resection of conceptus and hysteroplasty | Y | N | NA |
| SHAN SU et al. (2017) | 39 | 34 | G2P1; NA | 5 weeks, 5days | postoperative pathology | the left uterine wall; 5.1 | methotrexate and laparotomy excision | N | N | NA |
| Deanne Vagg et al. (2018) | 40 | 34 | P2; open myomectomy | 12 weeks | TVU; MRI | the myometrium of the right uterine cornua; 8 | TAH+BS | N | N | NA |
| Tatsuya Ishiguro et al. (2018) | 41 | 40 | P0; two laparoscopic myomectomy | 8 weeks | TVU | the subserosal site of the myomectomy scar; 1.6 | laparoscopic surgery | N | N | NA |
| Qi Zhang et al. (2019) | 42 | 30 | G1P1; cesarean section | 10weeks | TVU | posterior wall; 4 | laparoscopic surgery; injected methotrexate 50mg | N | N | NA |
| Juliane Nees et al. (2020) | 43 | 24 | G2P1; cesarean section | 8 + 4 weeks | US | intramurally near the left tubal os; 4.3 | laparotomy and lesion wedge resection | N | N | NA |
| Zhaojun Shen et al. (2020) | 44 | 20 | G2A1; curettage | 7weeks+ | US | left uterine wall; 3.1*2.6*2.9 | hysteroscopic surgery | N | N | Normal pregnancy after 2y and half month |
|  | 45 | 41 | G5P2A3; curettage*3; cesarean section | 8weeks+ | postoperative | right uterine wall near cornu; 2.7*2.7*2.6 | laparoscopic surgery + hysteroscopy | N | N | not sexually active |
|  | 46 | 30 | G1A1; curettage; laparoscopic + hysteroscopy | ET 4+w | US | right posterior uterine wall near fundus; 2.2 | laparoscopic surgery | N | N | no need for fertility |
|  | 47 | 36 | G3P1A2; Curettage; cesarean section | ET 4+w | postoperative | right uterine wall; 1.2 | laparoscopic surgery | N | N | term delivery by CS after IVF |
|  | 48 | 26 | G2A1; right hornectomy + salpingectomy | 5+w | postoperative | right uterine fundus near cornu; 1.4*1.3*1.1 | laparoscopic surgery | N | N | one failed IVF |
|  | 49 | 37 | G2A2; curettage twice | 22w after curettage | postoperative | right uterine wall near fundus; 2.9*2.7*2.4 | laparoscopic surgery + hysteroscopy | N | N | preparing IVF |
|  | 50 | 39 | G2P1A1; curettage; CS | 7+w | postoperative | right uterine fundus near cornu; 1.7 | laparoscopic surgery + hysteroscopy | N | N | no need for fertility |
|  | 51 | 31 | G1A1; curettage; hysteroscopy*2 | 9+2 | postoperative | right anterior uterine wall; 3.5*3.8*2.3 | laparoscopic surgery + hysteroscopy | N | N | contraceptive being used |
| Michael Chaikof et al. (2020) | 52 | 35 | G2P2; curettage; hysteroscopy | 8weeks+ | US | the fundus of the uterus; NA | laparotomy and fundal hysterotomy, lesion removal | N | N | NA |
| Yuan Liu et al. (2020) | 53 | 28 | G2P1; right salpingectomy for EP; IVF for 2 times | 7weeks | intraoperative | the right posterior uterine wall; 1.4 | laparoscopic excision of conceptus | N | N | IVF-ET 5 months later, chemical pregnancy; 7 months, IVF-ET and recurrence of intramural pregnancy, again received surgical resection and hysteroplasty surgery under laparoscopy |
| Our research | 54 | 20 | G1P0; artificial abortion | 17weeks+2d | TVU | right posterior wall of the uterine, near the fundus; 14 | laparotomy, hysterotomy, conceptus enucleation, and hysteroplasty; abdominal aorta balloon occlusion | N | N | not fertility |
|  | 55 | 42 | G4P1A2; artificial abortion; induced abortion | 7weeks+ | TVU | left posterior wall of the uterine fundus; 2.7*1.0 | laparoscopic excision of conceptus | N | N | not fertility |
|  | 56 | 38 | G2P1; cesarean section | 10weeks+ | TVU | left posterior wall of the uterine, near the fundus; 6.1*4.5 | laparoscopic excision of conceptus; hysteroscopy | N | N | not fertility |

Abbreviations: EP, ectopic pregnancy; HCG, human chorionic gonadotrophin; US, ultrasound; TVU, transvaginal ultrasonography; MRI, magnetic resonance imaging; CS, cesarean section; TAH + BS, total abdominal hysterectomy and bilateral salpingectomy; IVF-ET, in vitro fertilization – embryo transfer; NA, not applicable.
