# Supplementary material for: Intramural Ectopic Pregnancy: Clinical Characteristics, Risk Factors for Uterine Rupture and Hysterectomy
Source: Front Med (Lausanne). 2021 Oct 28;8:769627. doi: 10.3389/fmed.2021.769627 (PMC8583088; doi:10.3389/fmed.2021.769627)
Supplement: Supplementary Table 2 — (a) Univariate and multivariate analysis of clinical outcomes (uterine rupture vs. not rupture); (b) The predictive performance of the identified risk factors for uterine rupture. [file Table_2.DOCX]

**Table S2a** Univariate and multivariate analysis of clinical outcomes (uterine rupture vs. not rupture)

| Factors | Not rupture | Rupture | X^2^ | P value | Logistic regression analysis |
| --- | --- | --- | --- | --- | --- |
|  |  |  |  |  | OR (95%CI) P |
| Age (years) | 31.5 ± 6.9 | 28.7 ± 5.0 |  | 0.239 |  |
| Uterine surgery (<2 times/≥2times) | 23/19 | 4/4 | 0.092 | 1.000 |  |
| Methods of conception (nature/ART) | 41/6 | 8/1 | 0.019 | 1.000 |  |
| Serum β-HCG (IU/L)  Diagnosis (preoperative/intra or postoperative) | 18651.6  27/20 | 13557.0  4/5 | 0.517 | 0.703  0.493 |  |
| GA (≤10/>10, weeks) ^a^ | 36/9 | 3/6 | 8.142 | 0.010* | 8.000 1.456 - 43.966 0.017* |
| Location of GS (fundus/other) ^a^ | 11/36 | 6/3 | 6.687 | 0.017* | 7.000 1.271 – 38.543 0.025* |

**Table S2b**. The predictive performance of identified risk factors for uterine rupture.

| Factors | OR (95% CI) | P value | AUC (95% CI) | P value | AUC (95% CI, combination) | P value |
| --- | --- | --- | --- | --- | --- | --- |
|  |  |  |  |  | 0.793 (0.596 – 0.989) | 0.006 |
| GA (weeks) | 8.000 (1.670 – 38.323) | 0.009 | 0.733 (0.540 – 0.927) | 0.028 |  |  |
| Location of GS (fundus/other) | 6.545 (1.401 – 30.582) | 0.017 | 0.716 (0.523 – 0.910) | 0.041 |  |  |

Abbreviations: GA, gestational age; GS, gestational sac; HCG, human chorionic gonadotrophin; AUC, area under the curve.

*a* Factors applied to multivariate analysis; * *p* < 0.05*
