# Supplementary material for: Intramural Ectopic Pregnancy: Clinical Characteristics, Risk Factors for Uterine Rupture and Hysterectomy
Source: Front Med (Lausanne). 2021 Oct 28;8:769627. doi: 10.3389/fmed.2021.769627 (PMC8583088; doi:10.3389/fmed.2021.769627)
Supplement: Supplementary Table 3 — Univariate and multivariate analysis to identify the potential risk factors for hysterectomy. [file Table_3.DOCX]

**Table S3** Univariate and multivariate analysis of clinical outcomes (hysterectomy vs. not hysterectomy)

| Factors | No | Hysterectomy | X^2^ | P value | Logistic regression analysis |
| --- | --- | --- | --- | --- | --- |
|  |  |  |  |  | OR (95%CI) P |
| Age (years) | 30.9 ± 6.7 | 32.3 ± 6.4 |  | 0.592 |  |
| Uterine surgery (<2 times/≥2times) | 24/20 | 4/3 | 0.016 | 1.000 |  |
| Methods of conception (nature/ART) | 41/7 | 8/0 | 1.333 | 0.577 |  |
| Serum β-HCG (IU/L)  Diagnosis (preoperative/intra or postoperative) | 18702.1  27/21 | 1500.0  4/4 | 0.108 | 0.443  1.000 |  |
| GA (≤10/>10, weeks) ^a^ | 37/9 | 2/6 | 10.439 | 0.004* | 12.333 2.125 - 71.565 0.005* |
| Location of GS (fundus/other) | 14/34 | 3/5 | 0.225 | 0.688 |  |
| Uterine rupture (yes/no) | 5/43 | 4/4 | 7.965 | 0.017* |  |

The predictive performance of GA on hysterectomy: AUC 0.777 (95% CI: 0.590 – 0.965), P = 0.013.

Abbreviations: GA, gestational age; GS, gestational sac; HCG, human chorionic gonadotrophin; AUC, area under the curve.

*a* Factors applied to multivariate analysis; * *p* < 0.05*
